# Editorial: COVID-19 related acute vascular distress syndrome: from physiopathology to treatment

**DOI:** 10.3389/fmed.2023.1260309

**Published:** 2023-08-01

**Authors:** Yazine Mahjoub, Daniel Rodenstein, Vincent Jounieaux

**Affiliations:** ^1^Cardiac Vascular Thoracic and Respiratory Intensive Care Unit, Department of Anesthesia and Critical Care, Amiens University Medical Centre, Amiens, France; ^2^Pneumology Unit, Cliniques Universitaires Saint-Luc, Université Catholique de Louvain, Brussels, Belgium; ^3^Respiratory Department, Amiens University Medical Centre, Amiens, France

**Keywords:** COVID-19, SARS-CoV, AVDS, ARDS, intrapulmonary shunt, right ventricular function, ACE-2, dexamethasone

In the lungs, COVID-19, at least initially, is a vascular insult, leading to overperfusion of affected zones resulting in low VA/Q situation. These changes lead to hypoxia, and consequently to compensatory hyperventilation. Since CO_2_ excretion is not affected due to its much higher solubility and to the linear characteristics of the CO_2_ dissociation curve, hypocapnia develops, to a point where the respiratory centers do not respond to hypoxia anymore, resulting in the condition of hypocapnic hypoxia without respiratory distress that was termed “Happy Hypoxia”. This corresponds to what we have identified with the acronym AVDS (Acute Vascular Distress Syndrome) (1). Later on, when the situation worsens and not only the vessels but also the lung parenchyma is involved, the resulting condition can be identified with the usual ARDS (Acute Respiratory Distress Syndrome) (2). It is worth mentioning that other physiologic mechanisms, somehow falling in oblivion in recent years, may also contribute to the phenomenon of “Happy hypoxia” (3).

Indeed, for us, COVID-19 patients, at any stages of their disease, are characterized by an increased pulmonary blood flow with intrapulmonary right to left shunt associated with alveolar injury of variable severity (2).

Nine articles have been accepted in this Research Topic that refers to different axes:

## 1. Physiopathology and risk factors

In their review, Cousin et al. have emphasized the role of angiotensin converting enzyme 2 (ACE_2_) in the pathophysiology of SARS-CoV-2 infection. ACE_2_ is widely expressed in lung vascular cells especially endothelial cells (composing the vessels structure) and pericytes (responsible of microvascular tone). The ACE_2_ receptor permits the attachment of the virus. This ligation to the ACE_2_ receptor causes its internalization and down-regulates the SARS-CoV-2 cellular entry. In such a case, the decrease in ACE_2_ activity creates an imbalance that increases the level of angiotensin 2 which leads to the pro-inflammatory and pro-fibrotic situation responsible of the severity of COVID-19. Patients with risk factors of baseline increased level of ACE_2_ or ACE_2_ imbalance (i.e.: male, overweight, diabetes mellitus, hypertension, chronic heart failure, etc...) are particularly exposed to severe forms of COVID-19.

Moreover, Bonato et al. evaluated in cohort study of COPD patients the risk factors of COVID-19. They found that the cardio-metabolic conditions were risk factor for developing COVID-19 (Bonato et al.). Interestingly, respiratory abnormalities (DLCO, emphysema) did not increase the risk of COVID-19 infection but were related to clinical outcome.

## 2. Bronchial hypervascularization

Pathological studies focusing on lung vasculature in COVID-19 patients have shown ultrastructural damage to the endothelium, the presence of SARS-CoV-2 in endothelial cells and more importantly pulmonary angiogenesis with vascular dilatations (4). In this Research Topic, Jounieaux et al. have shown that such vascular dilatations also concern the bronchial vasculature. The authors used narrow band imaging (NBI) during bronchovideoscopy to unveil bronchial hypervascularization during COVID-19 infection. This bronchial hypervascularization can explain not only some unexplained hemoptysis observed in COVID-19 patients but also, in part, the specific intrapulmonary shunt that we described in this viral infection as AVDS.

## 3. Thrombosis and pulmonary embolism

COVID-19 vascular involvement is also due to the coagulation impairment associated with SARS-CoV-2 infection with a high risk of venous thrombosis and pulmonary embolism. Kutsogiannis et al. compared patients with COVID-19 related ARDS and patients with ARDS related to other cause. They found that COVID-19 patients had a significant higher incidence of pulmonary embolism. Moreover, the authors found that a high level of D-dimer is a good predictor of PE in these patients. This COVID-19 related coagulopathy seems to involve not only the systemic pulmonary circulation but also portal circulation. Hence, Kheyrandish et al. reported a case series of portal thrombosis following COVID-19 infection or vaccination.

## 4. Cardiac involvement

Motloch et al. performed a one-year follow-up of patients hospitalized for COVID-19 pneumonia. They found that cardiovascular biomarkers (vascular cells adhesion molecule 1, serum soluble suppression of tumorgenity-2 and high-sensitive troponine I) were associated with mortality. Especially, they found that high-sensitive troponine I, a specific cardiac biomarker, might predict post-discharge mortality. This study emphasizes not only the vascular involvement but also the cardiac injury associated with this disease. In ICU COVID-19 patients, Beyls et al. have performed echocardiography to evaluate right ventricular function. They found that acute cor pulmonale (ACP) was more frequent in this disease even in non-intubated patients. ACP was not related to pulmonary embolism in the majority of patients but appears to be an independent risk factor of mortality.

## 5. Treatments

The beneficial effects of corticosteroids for hospitalized COVID-19 patients have been extensively proven regardless of the severity of the disease (5). Reindl-Schwaighofer et al. studied the effect of corticosteroids on ACE_2_ and fibrin degradation on human volunteers. They showed that dexamethasone reduced ACE_2_ upregulation and intra-alveolar markers of fibrinolysis. These findings may explain its beneficial role in COVID-19 (Reindl-Schwaighofer et al.). Regarding ventilatory support, Luján et al. have carried out a narrative review of 74 studies published during the pandemic. They reported that high-flow oxygen therapy, prone positioning and non-invasive ventilation have been extensively used for hypoxemia which appeared refractory to conventional oxygen therapy (Luján et al.). To date, the optimal approach is still debated and individualized strategy is needed.

## 6. Conclusion

All these articles published in the Research Topic untitled “*COVID-19 related acute vascular distress syndrome: from physiopathology to treatment*” in Frontiers in Medicine are consistent with the hypothesis early evoked during the pandemic (1). COVID-19 is indeed a vascular disease in which lungs vasculature (pulmonary and bronchial vessels) is impaired leading to intrapulmonary shunt and hypoxemia. This specific vascular injury is difficult to observe because it is hidden by concomitant alveolar lesions.

## Author contributions

YM, DR, and VJ contributed in conducting the literature review and writing the manuscript. All authors have read and approved the final manuscript.
